# The Effects of Indigo Waste Silage Prepared with Additives on Feed Availability, Rumen Fermentation Patterns, Blood Metabolites, and Hematological Indices in Beef Cattle

**DOI:** 10.3390/vetsci11120588

**Published:** 2024-11-22

**Authors:** Nirawan Gunun, Chatchai Kaewpila, Waroon Khota, Wasana Phlaetita, Pongsatorn Gunun

**Affiliations:** 1 Department of Animal Science, Faculty of Technology and Engineering, Udon Thani Rajabhat University, Udon Thani 41000, Thailand; nirawan.gu@udru.ac.th; 2 Department of Animal Science, Faculty of Natural Resources, Rajamangala University of Technology Isan, Sakon Nakhon Campus, Phangkhon, Sakon Nakhon 47160, Thailand; chatchai.ka@rmuti.ac.th (C.K.); waroon.kh@rmuti.ac.th (W.K.); 3 Department of Plant Science, Faculty of Natural Resources, Rajamangala University of Technology Isan, Sakon Nakhon Campus, Phangkhon, Sakon Nakhon 47160, Thailand; wasana.ph@rmuti.ac.th

**Keywords:** indigo waste silage, additives, digestibility, rumen fermentation, blood parameters, beef cattle

## Abstract

The purpose of this study was to evaluate the impact of different additives in fermented indigo waste on feed availability, rumen fermentation patterns, blood chemistry, and hematology in beef cattle. Four male crossbred beef cattle with a body weight (BW) of 230 ± 14 kg and 25 months of age were used in a 4 × 4 Latin square design. The indigo waste was ensiled without additive (CON) and with calcium hydroxide (CH), molasses (M), or cellulase (C). The indigo waste silage was incorporated into a fermented total mixed ration (FTMR). The neutral detergent fiber (NDF) content was lower in C-treated silage. The feed intake did not alter among treatments (*p* > 0.05). The ensiled indigo waste with additives had no influence on nutrient digestibility (*p* > 0.05), whereas the ensiled indigo waste with M increased CP digestibility (*p* < 0.01). The rumen pH, ammonia-nitrogen (NH_3_-N), or volatile fatty acid (VFA) proportions did not change by additive (*p* > 0.05). However, adding M increased the acetate-to-propionate ratio (C2:C3) (*p* = 0.04). In addition, the total protein in the blood was higher in C-treated silage (*p* = 0.01). The homological indices did not change by additive (*p* > 0.05), except for lymphocytes, which decreased when M and C were added (*p* < 0.01). In conclusion, adding M and C to indigo waste silage could enhance its nutritional value and improve digestibility, blood chemistry, or health status in beef cattle.

## Simple Summary

Indigo plants are harvested for indigo dye processing, which also produces indigo waste as a by-product in a rainy season. Indigo waste contains a high crude protein content and could be used in animal feed. Ensiling is a processing method for ruminant feed that is effective in preserving nutritional value and has a controlled microbial fermentation process. The present work aims to study the effects of ensiled indigo waste with additives including calcium hydroxide, molasses, and cellulase on feed utilization, rumen fermentation characteristics, and blood parameters in beef cattle. The findings suggest that the present study provided evidence that ensiled indigo waste with molasses and cellulase improves the nutritive value of silage, digestibility, blood chemistry, and health status in beef cattle.

## 1. Introduction

The lack of roughage and protein feed sources limits the production of beef cattle in tropical countries [1,2]. Utilizing alternative feed resources is crucial for cost reduction and diversification of the ruminant diet [3]. The indigo plant, known as *Indigofera tinctoria* L., has become established in Africa and tropical Asia [4]. In Thailand, a variety of small-scale industries use indigo and traditional methods to make natural indigo dye for cotton fabrics [5]. Following the dye production process, the stem and leaves consist of the remaining indigo waste, which is about 237–261 kg/ha [6]. The indigo waste contains 19.5% crude protein (CP), 72.4% NDF [6], and 3488 kcal/kg DM gross energy (GE) [7]. Our previous studies showed that adding up to 20% of dried indigo waste in the diets did not impact the feed availability, rumen fermentation patterns, hematology, or immune functions of beef cattle [7]. A concentrate diet suitable for cattle production in the tropics incorporates 10% of dried indigo waste. Ruminants can effectively utilize indigo by-products as a source of protein and fiber while also contributing to environmental sustainability and reducing feed cost. However, Thailand harvests the majority of its indigo plants for indigo dye production during the rainy season. Hence, indigo waste is extremely difficult to sun-dry during this season; hence, ensiling is considered the optimal preservation method [6].

Silage is a suitable method of preserving forage for later use as ruminant feed [8]. Silage additives can be incorporated into the forage to enhance the efficiency of silage preservation at different stages, from fermentation to ruminant feeding [9]. Cellulase is widely used to treat silage due to its ability to breakdown structural carbohydrates into soluble sugars that serve as substrates for the growth of lactic acid bacteria (LAB) under anaerobic fermentation [10,11]. Molasses can provide easily fermentable sugars for LAB growth, which results in the production of lactic acid [12,13]. The alkaline agents, particularly calcium hydroxide, facilitated the cleavage of ester bonds between lignin and hemicellulose, leading to a decrease in the fiber content of silage and an improvement in feed utilization in ruminants [14,15]. Our earlier study found that treating indigo waste with CH, M, or C has potential to improve the quality of silage, in vitro degradability, and rumen fermentation characteristics [6]. Accordingly, in vivo confirmation is required for the data obtained from in vitro trials. However, there has never been any research on the various additives used for ensiling indigo waste in cattle. Consequently, the objective of this study is to examine the influence of CH-, M-, or C-ensiled indigo waste on the nutritive value, feed utilization, rumen fermentation patterns, blood chemistry, and hematological indices of beef cattle.

## 2. Materials and Methods

### 2.1. Ethical Procedure

This study’s methodologies and procedures were approved by Rajamangala University of Technology Isan’s animal care and use committee (approval number 46/2565).

### 2.2. Preparation of Indigo Waste Silage with Additives

Following the dye extraction process, we obtained fresh indigo waste from the Ban Don Koi weaving and indigo dyeing group in Pannanikom, Sakon Nakhon, Thailand. This waste was subsequently cut to a size of 2–3 cm. The indigo waste was prepared with no additive (CON), CH at 2% fresh matter (FM), M at 3% FM, and C at 0.4 g/kg FM. The indigo waste was sprayed with the additives, and they were then well mixed. After that, they were kept in plastic drums at room temperature. After 30 days of ensiling, indigo silage samples (4 plastic drums per treatment, 4 treatments) had their pH determined using a portable pH meter, to assess their chemical composition. 

### 2.3. Experimental Animals and Treatments

Four male crossbred cattle (Brahman × Thai native), aged 25 months and with an initial BW of 230 ± 14 kg, were randomly assigned using a 4 × 4 Latin square. The indigo waste was fermented with CON, CH, M, and C. The FTMR included the indigo waste silage, with or without an additive, at 20% DM (Table 1). The FTMR was mechanically mixed and stored in plastic drums at room temperature for 21 days before feeding. The cattle received the FTMR ad libitum at 08:00 h and 16:00 h, with the goal of achieving a 10% refusal rate on an as-fed basis. Individual cages were employed to house the cattle, and they were provided with clean water at all times. A total of four periods, each lasting for a period of 21 days, were used to carry out the experiment. During the first 14 days of the experiment, the adaptation to diet was observed, while feces were collected over the last 7 days. A 7-day switchover occurred between periods.

### 2.4. Data Collection and Sampling Procedures

The BW of each cattle was recorded at both the start and end of each period. Every morning, FTMR samples were weighed to determine the amount offered and refusals. Fresh fecal samples (about 500 g) of each cattle were collected using rectal sampling. Each animal’s feces were mixed together at the end of every period and stored in the refrigerator. The samples were dried at 60 °C and ground (1 mm screen). The amounts of dry matter (DM) and ash [16] were analyzed. The CP content was obtained using an N analyzer (828 Series, LECO, St. Joseph, MI, USA). The contents of NDF and acid detergent fiber (ADF) were assessed utilizing the fiber analyzer (ANKOM 200, ANKOM Technology, Macedon, NY, USA), in accordance with the methodology outlined by Van Soest et al. [17]. The assessment of nutritional digestibility was conducted by employing acid-insoluble ash (AIA) [18]. 

At the end of each period, stomach tubes collected approximately 200 mL of rumen fluid from each animal at 0 and 4 h after feeding. The first of the ruminal samples (about 100 mL) was eliminated to avoid saliva contamination. A portable pH meter was used to promptly investigate the samples after they were filtered via four layers of cheesecloth. Then, rumen fluid was centrifuged at 16,000 rpm for 15 min at 4 °C and then tested for NH_3_-N (Kjeltech Auto 1030 Analyzer, Tecator, Hoganiis, Sweden) [19] and for VFA using gas chromatography (GC 8890; Agilent Technologies Ltd., Santa Clara, CA, USA) [20].

On the last day of each period, we collected blood samples (10 mL) from the jugular vein (0 and 4 h after feeding). The use of these samples was divided into two parts. In the first part, blood samples were stored in serum tubes for the evaluation of hemoglobin, hematocrit, white blood cells (WBCs), neutrophils, lymphocytes, monocytes, and eosinophils with a hematology analyzer (Mindray BC-3000 Plus, Shenzhen, China). In the second part, blood samples were stored in tubes with EDTA in order to ensure that a chemical analyzer (Mindray BS-600, Shenzhen, Chaina) could measure the total protein, glucose, and cholesterol.

### 2.5. Statistical Analysis

The data variances were analyzed using the general linear model (GLM) in SAS software (Version 6.12) with a 4 × 4 Latin square design [21]. The model Yijk = μ + Mi + Aj + Pk + εijk was used to evaluate the data, where Yijk is the observation from treatment i, cattle j, and period k; μ is the overall mean; Mi is the mean effect of the treatments (i = 1–4); Aj is the mean effect of the cattle (j = 1–4); Pk is the mean effect of the periods (k = 1–4); and εijk is the residual error. Duncan’s new multiple range test (DMRT) was used to evaluate the treatment mean difference [22]. Significant differences were determined using *p* < 0.05.

## 3. Results

### 3.1. Chemical Composition of Diets

All FTMRs can be produced using local feed resources, which have a composition of 60.5–63.0% DM and 14.5–14.8% crude protein (CP) (Table 2). The feed costs of FTMRs containing indigo waste silage fermented with CON, CH, M, and C were 18.76, 19.04, 19.08, and 18.79 USD/100 kg FM, respectively. Additionally, ensiling silage with CON, CH, M, and C had feed costs of 0.86, 1.59, 1.71, and 0.93 USD/100 kg FM, respectively (Table 3). Indigo waste treated with M reduced the DM content (*p* < 0.01). When adding CH to the silage, the organic matter (OM) and CP decreased (*p* ≤ 0.02), while the ash content increased (*p* < 0.01). Adding C-treated silage decreased the NDF content (*p* = 0.02). The ensiled indigo waste with additives did not influence the ADF content (*p* > 0.05). The addition of CH had a higher pH in silage (*p* < 0.01).

### 3.2. Feed Intake and Digestibility

The ensiled indigo waste with additives did not affect the DM intake (*p* > 0.05) (Table 4). Treatments had no effect on the digestibility of nutrients (*p* > 0.05), but adding M increased the digestibility of CP (*p* < 0.01).

### 3.3. Rumen Fermentation

The levels of NH_3_-N, total VFA, and pH in the rumen at 0 and 4 h after feeding were not different between treatments (*p* > 0.05) (Table 5). All additives had no effect on the proportion of acetate (C2), propionate (C3), or butyrate (C4) (*p* > 0.05). However, adding M to indigo waste silage increased the C2:C3 at 4 h after feeding (*p* = 0.04).

### 3.4. Blood Chemistry and Hematological Parameters

The silage’s additives had no effect on blood levels of glucose or cholesterol (*p* > 0.05) (Table 6). Silage treated with C had a higher total protein before feeding (*p* = 0.01). The additives to silage had no influence on the levels of hematology (*p* > 0.05) (Table 7), whereas lymphocytes at 4 h after feeding were lower in beef cattle fed silage treated with M and C (*p* < 0.01).

## 4. Discussion

After 30 days of ensiling, the DM content of the indigo waste silage in group M was reduced. The moisture content of liquid molasses is 24.4–35.8% [23,24]. Adding M may increase the moisture content, which in turn reduces the DM content in an indigo waste silage. The addition of CH to indigo waste silage increased the content of ash and led to a lower OM content. Adding lime to silage produced a high concentration of alkaline chemicals, which led to an increase in pH. Furthermore, the indigo waste silage in this study had a CP content range of 23.0–26.1%, which was greater than our earlier study’s CP content range of 19.5–21.5% [6]. This result could be attributed to the collection of indigo waste from different areas, the harvest period, or the proportion of stems and leaves, all of which can affect the CP content in the silage. Our recent study found that CH-treated indigo waste silage increased the CP content [6]. However, adding CH to indigo waste silage lowers the CP content when compared with the CON in the current study. This study used small-scale silos (plastic drums) with a silage capacity of approximately 80 kg, whereas previous studies used vacuum bags containing 0.5 kg. This difference could potentially impact the nutritive value of the ruminant diet, particularly the CP content. For other reasons, adding lime to silage does not inhibit the growth of *Clostridium*. Instead, it increases the breakdown of proteins and amino acids into other substances, including NH_3_-N and volatile organic acids. Furthermore, previous research indicated that adding C groups reduces the NDF content in silage [25,26]. This agrees with the present study, with the lower NDF content by C-treated indigo waste silage. Cellulase hydrolyzed cellulose and hemicellulose to release soluble sugars [27], thereby reducing the NDF content in silage. The additives had no impact on the content of ADF. The results were similar to those of Gunun et al. [6], who reported that adding CH, M, or C, or a mix of them, had no effect on the amount of ADF in indigo waste silage. This may be attributable to the time required to induce the effects of any additive on plant cell walls.

The additives to silage had no effect on the DM intake in cattle. This is consistent with recent studies that found the M- and C-treated silage did not change the feed intake [28,29]. In contrast, adding CH and M to silage increased the DM intake in cows and cattle [14,30]. It is plausible that recent studies used ruminant-fed silages ad libitum and supplemented with concentrate diets, whereas this study included indigo waste silage in the FTMR, which might not impact the DM intake of cattle. The M-treated silage in the FTMR increased CP digestibility compared to the other treatments. Previous studies have confirmed that adding MO to Napier grass silage enhances CP digestibility in cows [30]. These findings have two plausible explanations. First, the addition of M may stimulate fermentation, resulting in lactic acid production and a drop in pH, which in turn inhibits the hydrolyzed CP in the silage. Therefore, feeding silage to cattle may increase their intake of CP and improve their digestibility. Second, molasses may provide energy that synchronizes with non-protein nitrogen (NPN) for microbial protein synthesis in the rumen, thereby improving nitrogen utilization and rumen fermentation efficiency [31,32]. In addition, previous investigations reported that the addition of M, C, and CH in grass and sugarcane bagasse silage increased the NDF and ADF digestibility in cows and cattle [14,30,33]. So et al. [28] found that adding 40% of C- and M-treated sugarcane bagasse to the total mixed ration (TMR) of dairy cows increased the fiber digestibility. However, the current study found that the additives to indigo waste silage had no effect on the NDF and ADF digestibility. It is plausible that previous studies used ruminant-fed silages ad libitum, while this study’s inclusion of indigo waste silage with additives at low levels (20%) in the FTMR may not have had an impact on the fiber digestibility in cattle. Another explanation is that different levels of additives in silage, types of forage, fecal collection methods, etc., might influence the NDF digestibility of animals.

Ensiling indigo waste with additives did not alter the pH of the rumen. This agreed with earlier studies that found that adding M, C, and calcium oxide to silage did not alter rumen pH [29,30,34]. After feeding, the rumen’s concentration of NH_3_-N increased [35,36]. Rumen microbes convert the majority of dietary CP to NH_3_-N, the primary source of nitrogen for microbial protein synthesis [37,38]. The amount of NH_3_-N at 0 and 4 h after feeding in all treatments ranged from 22.2 to 23.4 mg/dL and from 25.7 to 26.9 mg/dL, respectively. This is the optimal concentration (8.5–30 mg/dL) for the growth of rumen microbes [39,40]. The additives in fermented indigo waste silage did not influence the levels of NH_3_-N. In contrast, our previous research found a lower NH_3_-N when adding CH and C to indigo waste silage in an in vitro study [6]. This different result could be attributed to the use of different techniques (in vitro and in vivo), which could potentially affect the rumen fermentation patterns.

The rumen’s VFAs are considered to be a major energy source for ruminants, contributing mostly 70–80% of their requirement for energy [41,42]. The concentrations of VFAs are greatly influenced by the sampling time [43]. Before feeding, the levels of total VFA in the cattle were lower than after feeding in our study. In addition, the content of total VFA was not affected by silage additives. In contrast, the C- and M-treated silage increased the total VFA concentration in an in vitro study [44,45]. Our prior in vitro research found that the amount of rumen total VFA was lowest in group C, while it was highest in group M of indigo waste silage [6]. In a recent in vitro study, forage was ensiled with additives, whereas the present study used indigo waste silage with additives at 20% in the FTMR fed to cattle, which may have a different effect on the total production of VFA in the rumen. The proportion of VFA in the rumen is impacted by additives, or types of forage [46,47]. Previous investigations reported that adding M with C and CH to silage reduced the proportion of C2, while increasing C3 in the rumen of cattle [14,29]. Gunun et al. [6] reported that the use of M and C to ensile indigo waste led to higher levels of C3 and C4, but lower levels of C2 and C2:C3 in an in vitro study. Contrary to this study, the VFA production in the rumen did not change in cattle fed all additive-treated indigo waste silage. However, the addition of M at 4 h after feeding resulted in an increase in C2:C3 levels. These could be caused by the addition of M to silage, which raised the proportion of C2 while decreasing the C3; nevertheless, the change was not statistically significant, leading to greater levels of C2:C3 in the rumen of the cattle.

The rumen’s C3 has a correlation with the ruminant’s glucose levels. Rumen microbes produce C3, a fermentation substrate of carbohydrate, which is a major substrate for glucose synthesis in the liver [48]. The additions to the indigo waste silage had no effect on C3, which in turn did not alter the glucose concentration of the cattle. The addition of C-treated silage increased the total protein in the cattle’s blood. This result is probably due to ensiled C groups increasing protein utilization in cattle, which led to increased protein in the blood. However, this mechanism is unclear because the addition of C groups did not affect the chemical composition of silage, digestibility, or NH_3_-N concentration. The concentration of total protein in all treatments ranged from 6.4 to 6.6 g/dL, which was within the normal range (5.7 to 8.1 g/dL) for cattle [49].

Hematological analysis is commonly used to examine the health and nutrition as well as the source of abnormalities or malfunctions in cattle [50]. When silage was fermented with additives, the hematological parameters (hemoglobin, hematocrit, WBCs, neutrophils, monocytes, and eosinophils) remained unchanged. The amounts of hemoglobin, WBCs [50], hematocrit [51], neutrophils, monocytes [52], and eosinophils [53] in ruminant blood are within the usual range, according to previous research. Lymphocytes are responsible for the incredible specificity of adaptive immune responses [54,55]. In the present study, using M- or C-treated silage resulted in lower lymphocyte concentrations. This result is possible because M and C improved the quality of the silage by increasing the lactic acid bacteria, leading to higher levels of lactic acid and a lower pH [6], which in turn inhibited the growth of pathogens. This may cause a decrease in lymphocytes in the cattle’s blood, indicating improved health. Moreover, beef cattle had a normal lymphocyte concentration (43.5–57.8%) in comparison to prior studies (43.2–66.3%) [52,56].

## 5. Conclusions

The C-treated indigo waste silage reduces the content of NDF, while the CH group increases the content of ash and pH silage. The additives did not influence the DM intake and nutrient digestibility, except for the CP digestibility, which increased with M-treated silage. The ruminal pH, NH_3_-N, and VFA production were not altered by any additive added to silage, while the C2:C3 was higher in M-treated silage. Furthermore, any additives did not change blood glucose or cholesterol, whereas total protein was higher in silage treated with C groups. The hematological indices had no effect on silage’s additives, except for lymphocyte concentrations, which were lower in M- and C-treated silage. In summary, the addition of M and C to indigo waste has the potential to enhance its nutritional value, CP digestibility, blood total protein, and health status by reducing lymphocytes, all without affecting the feed intake or rumen fermentation of beef cattle. To explore the effects of indigo waste silage with additives on beef cattle’s growth performance, additional research is required.

## Figures and Tables

**Table 1:** Ingredients of the fermented total mixed ration (FTMR) fed to cattle.

Item	CON	CH	M	C
Ingredient, % DM				
Rice straw	20.0	20.0	20.0	20.0
Indigo waste silage	20.0	20.0	20.0	20.0
Cassava chip	34.5	34.5	34.5	34.5
Soybean meal	10.0	10.0	10.0	10.0
Rice bran	10.0	10.0	10.0	10.0
Molasses	2.0	2.0	2.0	2.0
Urea	1.5	1.5	1.5	1.5
Mineral and vitamin mixture	1.0	1.0	1.0	1.0
Salt	0.5	0.5	0.5	0.5
Sulfur	0.5	0.5	0.5	0.5

**Table 2:** Chemical composition of the FTMR diets.

Item	CON	CH	M	C
Chemical composition				
Dry matter, %	60.5	63.0	62.2	61.4
Organic matter, % DM	89.8	87.8	89.0	89.5
Crude protein, % DM	14.6	14.5	14.8	14.6
Neutral detergent fiber, % DM	51.4	47.2	50.4	48.0
Acid detergent fiber, % DM	34.8	31.0	33.4	34.7
Ash, % DM	10.2	12.2	11.0	10.5
Total feed costs (USD/100 kg fresh matter)	18.76	19.04	19.08	18.79

**Table 3:** Chemical composition and silage pH of indigo waste treated with different additives.

Item	CON	CH	M	C	SEM	*p*-Value
Chemical composition						
Dry matter, %	27.5 ^a^	27.5 ^a^	21.9 ^b^	25.7 ^a^	0.70	<0.01
Organic matter, % DM	92.3 ^a^	85.3 ^b^	92.0 ^a^	91.4 ^a^	0.43	<0.01
Crude protein, % DM	26.1 ^a^	23.0 ^b^	24.7 ^ab^	25.6 ^a^	0.62	0.02
Neutral detergent fiber, % DM	70.4 ^a^	68.2 ^a^	66.5 ^ab^	60.7 ^b^	1.87	0.02
Acid detergent fiber, % DM	54.3	53.8	52.9	52.5	1.84	0.89
Ash, % DM	7.7 ^a^	14.7 ^b^	8.0 ^a^	8.6 ^a^	0.42	<0.01
pH	5.2 ^a^	7.4 ^b^	5.0 ^a^	5.2 ^a^	0.11	<0.01
Feed costs (USD/100 kg fresh matter)	0.86	1.59	1.71	0.93	-	-

^a,b^ Values on the same row with different superscripts differed (*p* < 0.05).

**Table 4:** Effect of ensiled indigo waste with additives on feed intake and digestibility in beef cattle.

Items	CON	CH	M	C	SEM	*p*-Value
Dry matter intake						
kg/d	4.2	4.2	4.1	4.3	0.07	0.31
%BW	1.8	1.8	1.8	1.8	0.01	0.57
Digestibility coefficients, %						
Dry matter	62.1	63.2	66.1	64.0	1.44	0.34
Organic matter	65.1	65.3	68.6	66.6	1.36	0.35
Crude protein	61.5 ^ab^	56.3 ^b^	69.7 ^c^	61.8 ^a^	1.53	<0.01
Neutral detergent fiber	43.9	42.1	47.5	42.1	2.15	0.33
Acid detergent fiber	31.6	31.0	35.6	34.6	2.15	0.42

^a,b,c^ Values on the same row with different superscripts differed (*p* < 0.05).

**Table 5:** Effect of ensiled indigo waste with additives on rumen fermentation in beef cattle.

Items	Hours After Feeding	CON	CH	M	C	SEM	*p*-Value
Rumen pH							
	0	7.1	7.1	7.0	7.1	0.06	0.81
	4	6.9	7.0	6.9	7.0	0.08	0.67
NH_3_-N, mg/dL							
	0	22.8	23.4	23.4	22.2	1.10	0.85
	4	26.9	25.7	25.7	25.7	1.62	0.93
Total VFA, mmol/d							
	0	71.1	73.0	74.8	83.5	9.91	0.82
	4	93.5	77.3	86.7	75.2	13.6	0.78
VFA, mol/100 mol							
Acetate (C2)							
	0	65.3	67.2	65.4	64.6	1.71	0.76
	4	62.5	63.8	67.3	64.7	1.24	0.14
Propionate (C3)							
	0	17.5	16.2	15.1	16.7	0.91	0.38
	4	19.8	18.6	15.9	16.6	1.15	0.16
Butyrate (C4)							
	0	12.2	11.9	14.4	13.6	1.12	0.43
	4	13.6	13.5	12.6	14.1	1.39	0.89
Iso-butyrate (i-C4)							
	0	1.6	1.6	1.6	1.7	0.15	0.96
	4	1.4	1.5	1.1	1.6	0.18	0.37
Valerate (C5)							
	0	1.6	1.1	1.4	1.3	0.34	0.69
	4	1.4	1.2	1.6	1.3	0.22	0.78
Iso-valerate (i-C5)							
	0	1.8	2.0	2.1	2.1	0.14	0.42
	4	1.3	1.4	1.5	1.7	0.13	0.34
C2:C3							
	0	3.8	4.2	4.4	3.9	0.28	0.54
	4	3.3 ^a^	3.4 ^a^	4.2 ^b^	3.9 ^ab^	0.20	0.04

^a,b^ Values on the same row with different superscripts differed (*p* < 0.05).

**Table 6:** Effect of ensiled indigo waste with additives on blood chemistry in beef cattle.

Items	Hours After Feeding	CON	CH	M	C	SEM	*p*-Value
Glucose, mg%							
	0	71.3	76.5	75.3	75.3	2.84	0.61
	4	80.0	76.5	78.3	75.5	1.74	0.36
Cholesterol, mg/dL							
	0	63.8	64.0	71.3	66.5	3.39	0.43
	4	65.5	65.8	71.8	66.0	3.71	0.61
Total Protein, g/dL							
	0	6.4 ^a^	6.3 ^a^	6.4 ^a^	6.6 ^b^	0.38	0.01
	4	6.5	6.4	6.4	6.5	0.07	0.52

^a,b^ Values on the same row with different superscripts differed (*p* < 0.05).

**Table 7:** Effect of ensiled indigo waste with additives on hematology in beef cattle.

Items	Hours After Feeding	CON	CH	M	C	SEM	*p*-Value
Hemoglobin, g/dL							
	0	11.1	10.6	11.4	11.4	0.26	0.18
	4	10.8	10.6	11.0	10.7	0.16	0.58
Hematocrit, %							
	0	33.3	32.3	34.0	34.5	0.70	0.22
	4	32.5	32.5	32.5	33.0	0.43	0.80
White blood cells, 10^9^/L							
	0	11.2	11.7	11.9	11.7	0.59	0.87
	4	13.4	13.4	14.3	13.9	0.67	0.76
Neutrophils, %							
	0	31.3	43.0	42.5	39.5	5.06	0.40
	4	37.3	39.5	44.8	40.8	2.36	0.25
Lymphocytes, %							
	0	57.8	45.8	43.3	46.0	5.55	0.34
	4	51.3 ^a^	50.0 ^a^	41.3 ^b^	43.5 ^b^	1.42	<0.01
Monocytes, %							
	0	6.3	4.0	6.0	6.3	1.15	0.49
	4	6.0	4.3	4.3	4.8	0.55	0.18
Eosinophils, %							
	0	3.3	3.5	6.8	5.0	0.89	0.10
	4	3.8	3.8	5.0	4.3	0.70	0.58

^a,b^ Values on the same row with different superscripts differed (*p* < 0.05).

## Data Availability

Data are contained within the article.
